# AFM Characterization of Halloysite Clay Nanocomposites’ Superficial Properties: Current State-of-the-Art and Perspectives

**DOI:** 10.3390/ma15103441

**Published:** 2022-05-10

**Authors:** Mariafrancesca Cascione, Valeria De Matteis, Francesca Persano, Stefano Leporatti

**Affiliations:** 1 Department of Mathematics & Physics “Ennio De Giorgi”, University of Salento, Via Arnesano, 73100 Lecce, Italy; valeria.dematteis@unisalento.it (V.D.M.); francesca.persano@unisalento.it (F.P.); 2 CNR Nanotec—Institute of Nanotechnology, Via Monteroni, 73100 Lecce, Italy

**Keywords:** atomic force microscopy, halloysite clay nanotubes, nanocomposites, surface properties

## Abstract

Natural halloysite clay nanotubes (HNTs) are versatile inorganic reinforcing materials for creating hybrid composites. Upon doping HNTs with polymers, coating, or loading them with bioactive molecules, the production of novel nanocomposites is possible, having specific features for several applications. To investigate HNTs composites nanostructures, AFM is a very powerful tool since it allows for performing nano-topographic and morpho-mechanical measurements in any environment (air or liquid) without treatment of samples, like electron microscopes require. In this review, we aimed to provide an overview of recent AFM investigations of HNTs and HNT nanocomposites for unveiling hidden characteristics inside them envisaging future perspectives for AFM as a smart device in nanomaterials characterization.

## 1. Introduction

Halloysite nanotubes (HNTs) are an inexpensive alternative similar to multiwalled carbon nanotubes (CNTs). HNTs are new 1D natural nanomaterials with a specific combination of tubular nanostructure, good biocompatibility, high mechanical strength, large aspect ratio, natural availability, and rich functionality. These characteristics create exceptional mechanical, thermal, and biological properties that are available at a low cost for HNTs–polymer nanocomposites [1,2]. Their nanostructured surface and chemical composition are usually characterized either by high resolution microscopy such as Scanning Electron microscopy (SEM) or Transmission Electron Microscopy (TEM) most often accompanied by spectroscopy techniques like Fourier Transform Infrared Spectroscopy (FTIR) to discern closer details. A powerful alternative is represented by Atomic Force Microscopy (AFM), which allows for characterizing topographic, adhesive, friction, roughness, and nanomechanical features by a raster-scanning sample surface [3].

With the rise of nanotechnology, the AFM represented a key instrument over the past 30 years [3]. The first AFM setup allowed for obtaining only topographic images in contact operation mode. Successively, the acquisition system has been greatly enhanced by the introduction of optical tools [4] able to reveal the nanometric deflection of a cantilever during the scan. This has not only simplified the acquisition procedure, but it also improved the resolution thanks to the capability to simultaneously record the contribution of van der Waals and friction force exerted on the AFM tip. Furthermore, the optical system has permitted the development of the tapping mode operation [5,6], in which the probe is excited at its resonance frequency while being scanned across the surface. During the surface scan, the probe oscillation parameters change punctually related to the morphology of sample surface, i.e., the sample–tip distance. These changes are compensated by a feedback loop system that keeps the amplitude or frequency shift at a fixed value. The compensating signal is used to obtain high-resolution images of the surface topography since lateral forces are removed. In addition, the cantilever exerts small forces (e.g., in 1nN range) against the sample; then, this operating mode allows for characterizing soft material without damaging it.

Currently, AFM is undoubtedly the most versatile and powerful tool to characterize sample surface at a nanoscale: unlike electronic microscopy or scanning tunneling microscopy, it does not require previous treatment of a sample, such as metallization. In addition, it allows for performing the measurements in any ambient condition (in air or in liquid). Furthermore, AFM permits obtaining information about the mechanical properties of the investigated material. Through analysis of the tip-sample contact mechanics, quantitative measurements such as adhesion, viscoelasticity elastic modulus, and shear modulus can be detected. In the last few years, significative improvements have been made by implementing imaging capabilities, tip preservation, and quantitative mechanics for multifrequency measurements, by adding new AFM modes like amplitude modulation and peak-force tapping [7]. Moreover, in an inspiring review, Garcia and Herruzo overviewed the development of five different modes of multifrequency force microscopy, focusing on applications in proteins, nanostructures, and cells paving the way to combine spatial, compositional sensitivity, and time resolution of materials in native environments and to access properties not measurable in conventional microscopies [8].

**Figure 1:**
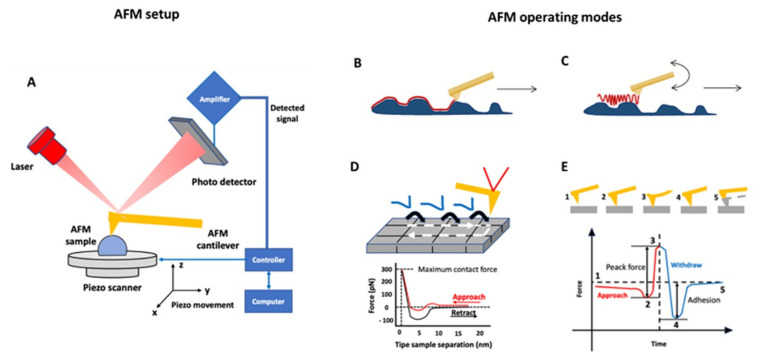
(**A**) AFM working principle: the probe, which includes a tip fixed to the free end of a flexible cantilever, scans the sample, and punctually interacts with its surface; this induces a change in cantilever deflection that is recorded by means of different techniques, mainly using the optical lever system. The processing signals generated by these interactions allows for analyzing local properties of the object. The principal AFM operating mode was schematically represented: (**B**) Contact and (**C**) Tapping Modes. These modes are essentially used to visualize at high resolution the topography of samples and to obtain information about superficial features. (**D**,**E**) represent force spectroscopy operation mode. Both allow for simultaneously collecting in a single scan topography and mechanical properties on the surface of organic or inorganic materials [9]. Quantitative Nanoscale Mechanical (QNM) can be carried out in non-resonance (i.e., contact) mode or in resonance mode (i.e., tapping). In detail: (**D**) Force Volume (FV) combines topographic acquisition in contact mode with repetitive indentation techniques in which Force−vs.−Distance curves were acquired; otherwise, (**E**) QNM combines topographic acquisition in tapping mode and Amplitude-vs.-Distance curves or Frequency-vs.-Distance curves recording. In (**E**) 1–5: Sequence of typical AFM cantilever cycling in QNM acquisition.

A comprehensive overview of these different modalities is reported in Figure 1. It represents the most used methods developed around AFM (Figure 1A) as a tool for morphological, physico-chemical, and nanomechanical study of HNT nanocomposites surfaces at high resolution. Contact Mode AFM (Figure 1B) was firstly overcome by Tapping Mode AFM (Figure 1C) to scan soft materials, whereas nanomechanical characterization by Force Volume (FV) (Figure 1D) microscopy allowed for assessment of (visco-) elasticity maps over scanned areas. AFM enabled researchers to separate topographical contributions into specific materials’ features. Furthermore, Tapping-QNM was also newly developed (Figure 1E), allowing for the coupling of soft tapping imaging together with mechanical quantification [9]. Novel scan assistant software/hardware features, available in Resonance -QNM Mode, now allows quick changing of scan parameters and fast scanning at unprecedent rates of imaging acquisition and also enabled monitoring on time very fast events eventually occurring at HNT nanocomposite surface, through environmental or external triggering.

## 2. Halloysite Nanotubes (HNTs) and HNT Nanocomposites

**Figure 2:**
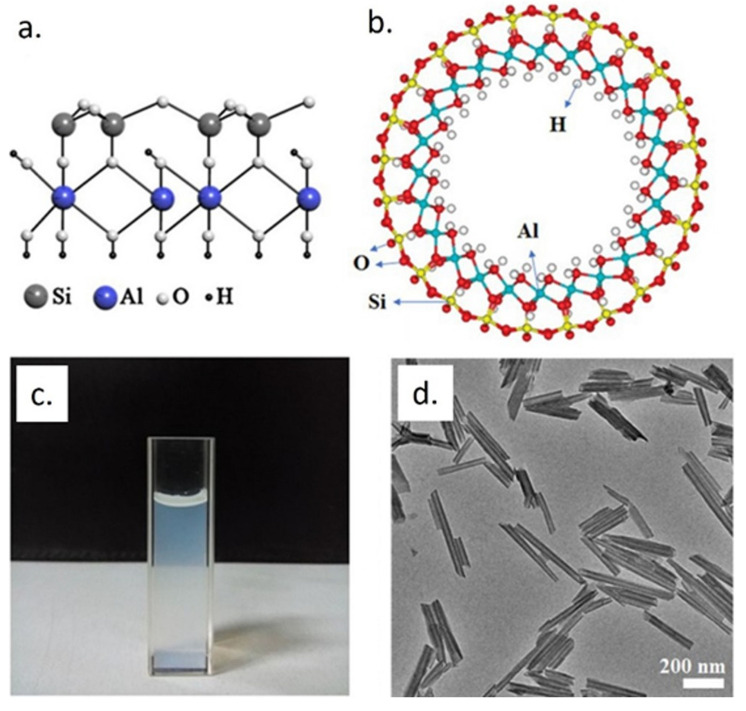
Schematic representation crystalline structure (**a**), cavity (**b**), water solution (**c**), and SEM acquisition of HNTs (**d**), adapted from [23].

Halloysite nanotubes (HNTs) are versatile nanomaterials characterized by a double layer of aluminum, silicon, hydrogen, and oxygen [10,11,12]. HNTs are derived from natural deposits all over the world, and their stoichiometry is Al_2_Si_2_O_5_(OH)_4_·2H_2_O with a ratio between alumina (Al) and silica (Si) of 1:1 [13] (e.g., see Figure 2). Typically, they have an inner diameter smaller than 100 nm and the length of HNTs varies from 0.5 to 1.5 µm [14,15].

A remarkable feature of halloysite is the different surface chemistry at the inner and outer sides of the tubes: the outer surface is made up of Si–O–Si, while the Al–OH groups are on the inner surface. This property permits functionalizing differently the surface and interior side of HNTs [16] by alkaline etching and grafting of nanoparticles, surfactants, polymers, and organosilanes [17,18,19].

In addition, it is possible to have different charges inside and outside the HNTs in a high pH range (i.e., between 3 and 8); in particular, the external surface is negatively charged while the interior has a positive charge [20] influencing their dispersion in aqueous solution and concentration, as well as on the presence of electrolytes. Besides the pH and charges, the electrolyte presence as well as the concentration is responsible for HNT dispersion [21]. In addition to these properties, the nanotubes have a cavity very suitable for the confinement of specific molecules, as it represents about 10% of its volume [22].

The internalization of specific molecules, even of large dimensions, allows their application in the biomedical field. Some drugs, namely nucleic acids, proteins, or carbohydrates [24,25,26,27,28,29,30], can be loaded by different methods (intercalation, adsorption, and tubular entrapment) in the HNT core [31]. This approach prevents the degradation of bioactive molecules and then permits delivering them to specific targets in the living organisms. These applications can be carried out due to the biocompatibility properties of HNTs [32]. The first biocompatibility experiments were performed on a halloysite coating assembled on a plastic or glass surface via the layer-by-layer process using cationic poly(ethyleneimine) or polylysine. HNTs are suitable nanofillers for polymers showing significant improvement in mechanical and thermal properties [33,34]. Several works investigated the mechanisms of the enhancement of mechanical properties in polymer–HNT nanocomposites including toughness, tensility, elastic moduli and flexural strength [35,36,37,38,39,40]. Then, since HNTs–polymer nanocomposites show good biocompatibility and drug release capabilities, they are therefore promising tools for tissue engineering and as drug vehicles [41]. Functionalized HNTs can also be used as adsorbents for environmental purification [42,43] modifying the halloysite surface [44].

## 3. AFM Investigations of HNT Nanocomposites

Among nano-clays, HTNs have been widely used in several application fields [7,41,45,46,47,48,49,50,51,52,53,54,55]. From the perspective of the aim of this mini-review, here we will carefully provide an overview of the most recent studies of HNTs, and HNTs- nano and bio-composites performed by the AFM technique. As previously introduced, the use of AFM applied to characterize these nanostructures is crucial, not only to visualize the nano-topographical features on the surface, but also to assess materials’ properties, including roughness, adhesion, and stiffness. In Table 1, we have briefly summarized recent AFM investigations in HNT-based nanocomposites with different operation modes, type of AFM used with specific limitations, and advantages in investigated nanocomposite samples.

AFM operating in contact mode was used by Liu and colleagues, characterizing morphological properties of synthetized chitosan/HNTs and alginate/HNTs bio-nanocomposite films [56,57]. The analysis of topographic acquisitions revealed how the addition of HNTs induced changes in the surface of chitosan at a nanoscale, leading to an increase of roughness both in chitosan and in alginate matrix. Because the increase of surface roughness improves the cell adhesion capability, these results suggested the improved performance of these nanocomposite materials for tissue engineering applications.

Mozia and co-authors [66] investigated the surface properties of HNT-modified polyethersulfone membranes obtained by means of a wet phase inversion method. Introduction of HNTs in the membrane significantly modified hydrophilicity and water permeability features of the composite membrane. Membranes were examined by AFM after drying them in ethanol. An increase in roughness correlated with increasing pH was detected upon HNT nanofiller loading. Furthermore, AFM images also confirmed enhancement of membrane permeability caused by pore increase, due to HNTs being added.

On the other hand, Zhang and co-workers produced nanocomposites to improve the hydrophilicity of the polyvinylidene fluoride membrane filling with HNTs and modified them with iron oxide, dopamine, and silane [67]. The membrane surface morphology and the pore structure were investigated by SEM and AFM. The authors detected differences in surface roughness and related them to membrane anti-fouling performances that could be used to efficiently treat oily wastewater.

In another interesting article, Liao et al. [68] investigate the effects of three different nanoclays, including HNTs, on properties of waterborne polyurethane matrix enriched with Jatropha oil. In detail, starting from the evidence that the addition of HNTs improves the polymeric material properties, but, in general, the introduction of clays into organic matrices is difficult due to their hydrophilicity, the authors evaluated the effects of Jatropha oil addition into the polyurethane matrix to improve the dispersion of clays. The results of average roughness, root-mean-square roughness, and maximum height parameters, obtained by the contact mode AFM topographies, suggest how the Jatropha oil enhances the compatibility between inorganic/organic phases of matrix and HNTs, respectively. It appears evident how estimation of roughness parameters is fundamental to characterize the reticulation step in nanocomposite materials. In several works, it is reported how this parameter is also strictly related to wettability, adhesion, and mechanical behavior of materials. For example, Gaaz and colleagues [69] investigated the properties of PVA-PVP composite materials enriched with modified HNTs by means of phosphoric acid and using malonic acid as a crosslinker. In this work, the AFM characterization of the roughness parameter played a key role in understanding the effects of different polymerization steps, on which the performance of material in dentistry applications depends.

PVA and PVP materials were largely investigated from the perspective of nanomedical applications, thanks to their biodegradability, low toxicity, and biocompatibility. Unfortunately, PVA/PVP nanocomposite films are highly hydrophilic and, consequently, they have fewer cell adhesion properties. In a very recent paper, Kouser et al. [59] investigated the roughness alteration of PVA/PVP bio-nanocomposite films after the addition of chitosan-modified HNTs. AFM investigation, performed in tapping mode in air condition, revealed homogenous dispersion of the modified HNTs and, consequently, a reduced roughness with respect to unmodified PVA/PVP film.

Once again, the tapping mode was used by Abdullah et al. [58] to assess ecofriendly bio-nanocomposite films of Polyvinyl Alcohol/Starch/Glycerol enriched with HNT at different weight percentages. By the analysis of topographic acquisitions, the aspect ratios of HNTs and the surface roughness of bio-nanocomposite films were achieved. These data in concert with results obtained by water vapor transmission and permeability investigations suggest that HNT addition modifies the roughness and in turn enhances the barrier properties of films, making them suitable for food packaging applications. Hatami et al. [60] prepared by sonochemical synthesis a novel type of hybrid nanocomposite that consisted of phenol-formaldehyde, polydopamine, and modified HNTs. By AFM investigations, the successful synthesis of NCs with good dispersion properties was confirmed. The authors have quantified the fractal dimension (D) (describing the self-similarity of the topographic profile on different scales, which reflects the complexity and irregularity of the surface) values by analysis of AFM acquisitions: the power spectrum, partitioning, and triangulation were applied to analyze D value from AFM statistics. Finally, the AFM investigation results indicated that the irregular surfaces of NCs comprise stands with dissimilar heights associated with the nano-tubular structure [60].

In a recent study, Liu et al. [70] fabricated eco-friendly and high-potential hybrid soy protein isolate (SPI)-based nanocomposites with improved tensile strength. The hybrid of carboxymethylated chitosan and HNTs was added to the SPI and 1,2,3-propanetriol-diglycidyl-ether solution, and all nanostructures were characterized by several techniques, including AFM. The latter was used to acquire in tapping mode the topographic and phase images of SPI-based films. The root-mean-square roughness evaluation suggested that the presence of nanofillers caused an increase in the surface roughness, probably due to the discontinuous filling of nanotubes.

**Figure 3:**
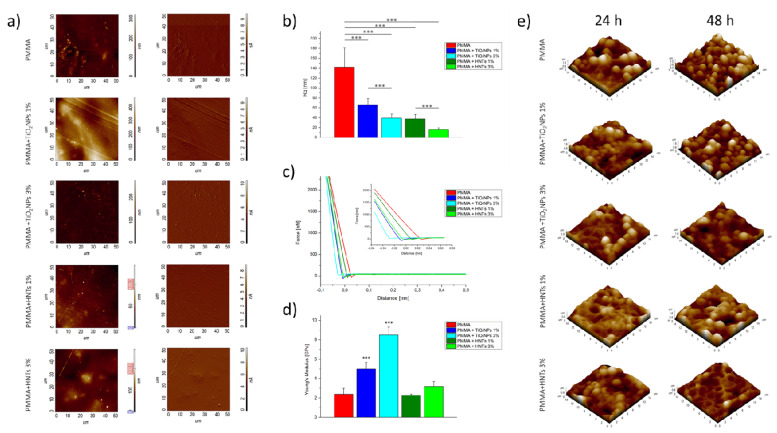
AFM analysis performed on PMMA/HNTs and PMMA/TiO_2_NPs nanocomposites. (**a**) topographical acquisitions in the height (left) and deflection (right) channel; (**b**) root main square roughness analysis (*p*-value < 0.005 ***); (**c**) representative nanoindentation curves (only approach data portion) acquired on different samples; and (**d**) Young’s modulus values analysis (*p*-value< 0.005 ***); (**e**) three−dimensional AFM topographical acquisitions, performed on different PMMA−based substrates after 24 and 48 h of *C. Albicans* colonization (adapted with permission from Ref. [64]).

In the past several years, AFM was also used to characterize material properties of HNTs/Polymethylmethacrylate (PMMA) nanocomposites, with the aim to investigate the potential improvements in PMMA material performance after HNT addition in different application fields. In particular, in the field of medical reconstructive medicine, this nanocomposite material appears to be particularly interesting: PMMA is already widely used for oral prostheses, although its mechanical performance needs to be improved. With this aim, Cascione et al. [64] used AFM to characterize PMMA enriched with HNTs or titanium dioxide nanoparticles (TiO_2_) at two different concentrations (1 and 3 wt%). The topographies of surface were performed in tapping mode in air (Figure 3a); their analysis shows that roughness value dramatically decreases after nanofiller addition, in particular, in the case of HNT inclusion (Figure 3b); this effect was dose manner dependent. In addition, the analysis of nanoindentation curves demonstrated that stiffness was strongly increased adding TiO_2_ and weakly when the PMMA was doped with HNTs (Figure 3c,d). Finally, the authors concluded that the inclusion of nano-engineered materials can enhance the performance of PMMA for its use in prosthetic implants by inhibiting adhesion and proliferation of microorganisms (Figure 3e).

**Figure 4:**
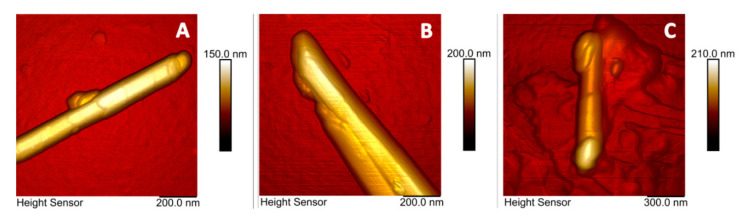
AFM topographic images of pristine (**A**), Mg (**B**), and Mg-DNA (**C**) modified halloysite nanotubes (adapted with permission from Ref. [63]).

Complexation of biopolymers with halloysite nanotubes (HNTs) can greatly influence their applicability as building blocks. To support this statement, AFM was used by Batasheva and co-workers [63] to investigate nanomechanical properties of native and Mg- and Mg-DNA modified halloysite clay nanotubes. They detected the presence of DNA on the nanotube surface through the changes in the surface adhesion force, analyzing the images carried out in Tapping mode (Figure 4): the rolled nature of HNTs topography and adhesive features are shown, allowing for demonstrating the presence of DNA bounded at their surface.

A nanoindentation method was also used in the experimental work carried out by Shi et al., in which the impact of the addition of different nanomaterials, including HNTs, on mechanical properties of epoxy coatings was examined [65].

The characterization of polymer membrane additivated with HNTs by means of AFM operating in Tapping QNM mode was firstly used by Naumenko et al. [71]. The authors investigated the morphomechanical properties enhancement of chitosan–agarose–gelatine matrix after HNT additivation, in order to design the implantable 3D cell scaffolds. Similar studies were performed by Kamal et al. [61] on polysulphone membrane doped with HNTs (0.2 and 5.0 wt%). In this work, operating in tapping-QNM in air conditioning and at room temperature, the membrane characterization was performed. The AFM topographies suggested the homogeneous distribution of HNTs within the membrane matrix additivated with 0.2 wt% HNTs. Moreover, the samples enriched with 5 wt% HNTs presented the cluster on the surfaces. These qualitative observations were confirmed by roughness quantification: the image analysis showed a significant reduction of the average roughness and root–mean–square roughness values in 0.2 wt% additivated samples. In addition, AFM in tapping-QNM mode was used to assess nano-mechanical properties of pristine and doped membranes, in terms of the elastic modulus and adhesion forces. The HNT addition caused a significant increase in Young’s Modulus value; this effect resulted in being higher in the sample doped with 0.2 wt% HNTs with respect to 0.5 wt%; this was probably due to excessive HNTs contents in the polysulphone matrix. In addition, the presence of aggregates was corroborated by adhesion mapping, which showed a remarkable heterogeneity with repulsive interactions prevailing at many sites on the membrane surface.

In a very recent paper, Cavallaro and co-workers proposed a novel keratin treatment of human hair by its aqueous mixtures with natural HNTs. In this case, the successful hair structure protection was also visually confirmed by AFM analysis. Tapping nanomechanical AFM in air was employed to discern morphology and nonspecific adhesion in pristine and keratin/halloysite hybrid-coated human hair. The AFM revealed the deposition of halloysite nanotubes, randomly positioned in a sort of dense monolayer on the cuticles. In addition, the keratin/halloysite hybrid-coated hair became stiffer and unveiled a higher nonspecific adhesions force, such as halloysite nanotubes coated with hydrolyzed keratin. These data further confirmed that the surface deposition of inorganic nanotubes enhance the mechanical stiffness of the native human hair, paving the way for novel hair care products and applications [60]. The tapping-QNM technique was also used in another recent study, performed by Pasbakhsh’s group [72]. They synthetized self-healing microcapsules of alginate modified with HNTs and coated with chitosan. AFM investigations were performed to visualize surface topography at the nanoscale, to quantify the roughness parameters and to assess the elastic modulus of the alginate-based microcapsules.

## 4. Conclusions and Outlook

In this review, we have briefly provided an overview of recent progress in nanomaterial characterization by HNTs-composites by means of Atomic Force Microscopy and related techniques. AFM was mainly employed as a complementary tool for investigating morphological, mechanical, and materials-related details not detectable in HNT nanocomposites by means of electron microscopies such as TEM and SEM. Such HNT bio and/or nano composites are novel materials showing improving mechanical strength and functional properties, which may be used, for example, in cosmetics, biomedicine, plant nutrition or dentistry applications. To understand mechanisms and study novel features created by assembling these hybrid nanostructures, AFM is a decisive tool not only for morpho or topographical investigations but also for specific chemical-physical properties monitoring/investigations and as a demonstration of successful nanocomposites assembly. Electron Microscopy imaging alone does not allow an in-depth three-dimensional analysis. Although AFM was originally a surface imaging technique, the newly developed methods (such as Tapping-QNM, for example) will allow for discovering hidden features inside HNTs bio or nano composites.

## 5. Perspectives

Fast progress in instrumentational developments in AFM has recently allowed for overcoming difficulties that researchers were usually facing in nanomaterials analysis and characterization, which previously represented a challenge for this technique. Several new AFM modalities/imaging modes just came on the market in the last few years (Tapping-QNM, Fast Imaging, Quantitative Imaging, etc.). In the area of hybrid clay nanocomposites, the combination of topographical and morphological imaging with chemical, nanomechanical, magnetic or electrical imaging implemented in novel Atomic Force Microscopes paves the way for their use as essential tools for high resolution nanostructure characterization.

## Figures and Tables

**Table 1:** AFM analysis of HNTs-nanocomposites with related AFM mode employed, AFM type used, and relative AFMs’ specific advantages and disadvantages.

HNT-Based Composite	AFM Modes	AFM Type	Limitations	Advantages	Reference
Chitosan/HNTs Bionanocomposites	Contact mode	Multimode Nanoscope IIIA (Bruker^®^, Billerica, MA, USA)	Bio nano composites Deformation upon Scanning	Analysis of Composites Roughness and Mechanical Properties	Liu et al., 2012 [56]
Alginate/(HNTs) composite scaffolds	Contact mode	Multimode Nanoscope IIIA (Bruker^®^)	Composites Deformation upon Scanning	Analysis of Topography, Surface Roughness, and Interaction Properties	Liu et al., 2015 [57]
Polyvinyl Alcohol (PVA)/Starch (ST)/Glycerol (GL)/HNTs Bionanocomposite Films	Tapping mode	Dimension Fast Scan (Bruker^®^);	Composites Aggregation	Inspection of HNTs Aspect Ratio correlated to Composites Permeability Model	Abdullah et al., 2019 [58]
PVA/PVP/HNTs Bionanocomposite Films	Tapping mode	FLEX-AXIOM AFM (Nano surf^®^ Easy Scan 2, Lisstaal, Switzerland);	Bio composites dispersion aggregation	Investigation of Mechanical, Roughness and Thermal Properties	Kouser et al., 2022 [59]
NTs/PDA/PF Nanocomposites	Tapping Mode	Multimode Compact Frame (Bruker^®^);	Nanocomposite aggregation	Analysis of Nano Topography and Roughness	Hatami et al., 2020 [60]
Polysulfone/HNTs	Tapping QNM	Dimension Icon (Bruker^®^);	Sample Porosity	Inspection of Adhesion, Roughness, and HNTs Distribution in the Matrix	Kamal et al., 2020 [61]
Keratin/HNTs	Tapping QNM	Dimension Icon (Bruker^®^);	HNT Stability/Adhesion during Scanning	Investigation of Mechanical, and Adhesive Properties	Cavallaro et al., 2020 [62]
Mg, and Mg-DNA HNTs	Tapping QNM	Dimension Icon (Bruker^®^);	HNTs-DNA binding in the Lumen vs. Exterior Surface Undetectable	Analysis of Surface Adhesive and Mechanical Properties	Batasheva et al., 2020 [63]
PMMA/HNTs	Semi Contact Mode	AFM INTEGRA (NT-MDT^®^ Spectrum Instr., Moscow, Russia)	HNTs -PMMA Adhesion/Coating	Analysis of Surface Topography, and quantification of Roughness and Young Modulus	Cascione et al., 2021 [64]
Epoxy/HNTs	Force vs. Distance Curves	Multimode Nanoscope IIIA (Bruker^®^);	Corrosion Resistance and Adhesion	Investigation of Mechanical Properties	Shi et al., 2009 [65]

## Data Availability

Not applicable.
